# From immune dysregulation to renal injury: mechanistic insights and translational opportunities in immune-mediated nephropathies

**DOI:** 10.3389/fcell.2026.1878787

**Published:** 2026-08-24

**Authors:** Xiaoman Wang

**Affiliations:** First People’s Hospital of Shangqiu, Shangqiu, China

**Keywords:** autoantibodies, autoimmune nephritis, immune tolerance, immunopathology, lupus nephritis, nephropathy

## Abstract

**Introduction and aim:**

Immune-mediated nephropathies are a mechanistically diverse group of kidney disorders in which dysregulated innate and adaptive immunity drive sustained inflammation, tissue injury, and progressive fibrosis. The kidney is not a passive target of these processes but an active immunological organ, where crosstalk between resident parenchymal cells and infiltrating leukocytes shapes both initiation and progression of disease. Advances in immunogenetics and molecular pathology have allowed several previously idiopathic entities—lupus nephritis, IgA nephropathy, ANCA-associated vasculitis, membranous nephropathy, and complement-mediated glomerulopathies—to be reclassified along mechanism-based lines. This review synthesizes the current understanding of their immunopathology, with emphasis on convergent signaling networks and on translational implications for diagnosis, risk stratification, and targeted therapy.

**Methodology:**

We adopted an evidence-based narrative approach, integrating clinical and experimental findings from immunology, nephrology, immunogenetics, and multi-omics. Key domains examined included immune tolerance, immune-complex biology, complement dysregulation, the renal immune microenvironment, cytokine and chemokine networks, non-coding RNA regulation, and immune-derived renal biomarkers. Comparative analysis was used to distinguish shared immune programs from disease-specific cascades across the major nephritic syndromes.

**Results and Discussion:**

Synthesizing evidence from over 150 peer-reviewed publications (2015–2025) across five major immune-mediated nephropathies, several recurrent immune modules emerge: autoantibody generation, complement activation, macrophage M1/M2 repolarization, and NLRP3 inflammasome priming. These converge on canonical signaling axes (TLR–MyD88, NF-κB, JAK–STAT, cGAS–STING, TGF-β/Smad), linking immune activation to glomerular injury and interstitial fibrosis. Established biomarkers (anti-dsDNA, anti-PLA2R, Gd-IgA1) guide diagnosis and risk stratification, while lncRNA signatures and exosomal cargo represent emerging non-invasive monitoring tools. Mechanism-targeted therapies—B-cell depletion, complement inhibition, and inflammasome modulation—are reshaping the treatment landscape.

**Conclusion:**

Immune-mediated nephropathies arise from the interaction between systemic immune dysregulation and a nephron-specific immune microenvironment. Distinguishing the shared immune programs from disease-defining cascades provides a rational basis for stratification and precision medicine. Integrated serologic, urinary, histologic, genetic, and multi-omics biomarkers can support earlier diagnosis and dynamic treatment adjustment. Therapy that targets the dominant pathogenic axis in each patient—rather than broad immunosuppression—offers the most realistic route to delaying end-stage renal disease, reducing toxicity, and improving long-term outcomes.

## 1 Introduction

The immune system plays a key role in protecting the body against foreign agents and is a primary determinant of various non-infectious diseases. Thus, Immunology extends beyond the “study of infections” and is now recognized as a basis of contemporary medicine. Immunologically driven innovations, from vaccination and transplantation to cellular and immunomolecule-based immunotherapies, have continued to enable identification, timely prevention, and precise management of both infectious and non-infectious diseases (Delves et al., 2000; Abbas et al., 2015; Dorak, 2002; Liu et al., 2025). However, dysregulated immune responses (Li D. et al., 2025), whether overactivation or misdirection, lead to immunopathology, which is manifested as immune-mediated tissue damage, chronic inflammation, fibrosis, or autoimmunity. The immune system specifically recognizes pathogens, activates targeted effector cells that selectively destroy infected cells and generates memory cells for future encounters. This cascade of events that normally protects against disease, when dysregulated, fails to differentiate, target and damage own tissues, thereby leading to autoimmunity (Akbarzadeh, 2025; Paul, 2011; Granoff and Webster, 1999; Yasmeen et al., 2024).

The importance of immunological processes has increasingly been revealed in an extensive spectrum of clinical disorders such as renal disease. The human kidney is both a passive target of the systemic immune dysfunctions and an effective participant in immune homeostasis, modulated by the local microenvironment, where infiltrating immune cells communicate, thus, regulating injury, repair, and fibrosis (Arneth, 2025; Ghara et al., 2025; Chao et al., 2025; Tecklenborg et al., 2018; Wang Y. et al., 2025). The renal system itself is constantly subject to immune complexes and complement activation; however, its structural and functional integrity relies on systematically controlled immune homeostasis (Petr and Thurman, 2023; Sinha and Nicholas, 2023). Recent developments in the medical field have shifted the diagnosis and treatment of nephropathy toward immunology-based treatment strategies. Improved understanding of the complement biology, autoantibodies characterization and immune checkpoint cascades has redefined the classification of glomerular diseases (Reddy, 2023; Mastellos et al., 2024). Clinical conditions once labelled as idiopathic nephritis are now recognized as distinct immunopathological diseases, including lupus nephritis (LN), IgA nephropathy (IgAN), ANCA-associated vasculitis (AAV), primary membranous nephropathy (MN), and complement-mediated glomerulopathies (CMG) (Kurts et al., 2025; Makker and Tramontano, 2011). These conditions show specific patterns of immunological responses, such as immune complex accumulation, complement system dysregulation, and pathogen- or antigen-driven autoreactivity; however, they share downstream cascades and mechanisms, including endothelial activation, podocyte injury, immune cell infiltration, and fibrosis (Ronco et al., 2021; Long et al., 2023; Yang et al., 2023).

The global impact of immune-mediated renal pathologies is significantly evident, imposing financial and psychological burden on patients and their families and huge pressure on the healthcare system. Approximately 850 million individuals worldwide are living with chronic kidney disease (CKD), with immune-mediated pathologies accounting for about 10%–20% of cases (Kurts et al., 2025; Guo et al., 2025a). Evidence shows that up to 60% of systemic lupus erythematosus (SLE) patients develop LN, a leading cause of end-stage renal disease (ESRD), particularly among women of reproductive age. Similarly, a progressive increase in the incidence of AAV has been reported, with a significantly higher proportion of older individuals (Mejia-Vilet et al., 2023; Mahajan et al., 2020). IgAN, characterized by IgA deposition in the kidneys, contributes up to 40% of primary glomerulonephritis in Asia. Furthermore, kidney diseases caused by CMG, once considered rare kidney pathologies, are now frequently identified through advanced genomics of complement system-related genes (Suzuki and Novak, 2024; Knoppova et al., 2016). These renal conditions also have a significant socioeconomic burden and challenges, which comprise long-term dialysis, transplantation, high cost of hospitalization, and immunosuppressive toxicity (Jadoul et al., 2024; Guo et al., 2025b; Lin, 2023). Owing to the rising global impact of chronic renal diseases, such as chronic kidney disease (CKD), acute kidney injury (AKI), and glomerular nephritis, measuring nephropathy using an immunological lens is not an option but essential to comprehending long-term prognostic results. Understanding how innate and adaptive immune systems interact with nephropathy can improve early diagnosis, risk assessment, targeted treatment, and management of disease progression (Qin et al., 2024; Wang L. et al., 2025).

Immunological responses in nephropathy can be explained three interconnected dimensions: physiological kidney-immune interactions, mechanisms of immune dysregulation that promotes nephropathies, and the translational and therapeutic implications that can guide current and emerging interventional approaches to shape long-term renal outcomes. A comparative analysis of these immune frameworks is summarized in Table 1. A mechanistic understanding of immune-mediated nephropathies requires first delineating the physiological kidney-immune interface that governs renal immune homeostasis.

**TABLE 1:** Immune system and nephropathy: A comparison of innate and adaptive cascades (Anders, 2013).

Characteristics	Innate immunity	Adaptive immunity
Recognition receptors (R)	Complement-R	B cell receptors
	Mannose-R	T cell receptors
	Toll-like-R	MHC I
	Inflammasomes	MHCII
	Low-affinity immunoglobulins	High-affinity IgG
Receptor clonality	Non-clonal	clonal
Receptor genes	Single gene, no rearrangement required	Encoded in gene segments, Rearrangement is required
Receptor agonists	Molecular patterns	Antigenic epitopes
Time delay of response	Immediate	Delayed
Effector mechanism	Opsonization, phagocytosis, granuloma formation, leukocyte recruitment, complement lysis, inflammation, and healing responses	Clonal expansion of antigen-specific B and T cells, antigen-specific immunoglobulins
Pragmatic kidney diseases	Urinary tract infection, acute kidney injury, glomerulosclerosis, tubulointerstitial fibrosis, crystal nephropathies, C3 glomerulopathy	IC glomerulonephritis, allograft rejection, interstitial nephritis

Collectively, immune dysregulation in nephropathies involves coordinated interactions between innate and adaptive immune components, resulting in sustained inflammatory responses and tissue damage. Instead of isolated action, these mechanisms form an interconnected network that highlights disease initiation and progression.

### 1.1 The kidney-immune system interfaces in health

The immune system is a dynamic surveillance and homeostatic network that constantly scans, adapts and communicates with local tissue microenvironments. Within the kidney, this immunonetwork is more actively involved in facilitating and maintaining host defense, immune homeostasis, and tissue-specific immunity. Kidney is one of the organs that is constantly exposed to blood-borne pathogen, immune complexes and a broad spectrum of complement activation products. The specific cellular and molecular mechanisms by which the kidney participates in immune responses—including cytokine and chemokine networks, complement activation, and crosstalk among resident and infiltrating immune cells—are discussed in detail in Section 3. Likewise, during homeostasis of renal tissues, the kidney facilitates immune regulation by eliminating immune complexes, regulating complement components and activity, and maintaining systemic volume and fluid-electrolyte balance, thereby indirectly affecting immune activation and the occurrence of inflammatory events or responses. Hence, it is possible that dysfunctions in these regulatory cascades contribute to the susceptibility of immune-mediated damage and inflammation and eventually to chronic nephritis (Tecklenborg et al., 2018; Foresto-Neto et al., 2024).

Also, tissue-specific immunity is critical in maintaining systemic health of the body through the regulation of the movement of the immune cells within distinct tissue microenvironments. The renal tissue microenvironment possesses unique structural and functional characteristics that are essential in the immunity and surveillance of the kidney. These include the presence of certain glomerular basement membrane, mesangial matrix, peritubular capillary network, and interstitial fibroblasts, which collectively modulate kidney-tissue-specific immune cell trafficking, complement complex control, repair pathways, and the transition from injury to fibrosis. These specific features differentiate renal immune responses from those in other tissues, such as the lungs or skin (Anders and Ryu, 2011; Kuppe, 2021; Li J. et al., 2025). Hence, understanding these tissue- or organ-specific immunological events is particularly essential, as studying “the immune system in general or in isolation” might not guide us on what is unique to the behavior or immunobiology of the kidney.

Overall, the kidney is an immunologically active organ which, beyond general protection, imparts homeostasis by communicating with innate immune system components, various immune cells, complement pathways and complexes, and adaptive responses. Therefore, disruption of this baseline homeostasis in renal tissues readily leads to immunological nephropathies and progressive organ dysfunction.

### 1.2 Immune dysregulation: the cause of nephropathy

A dysregulated immune system in the kidney leads to a range of severe complications, from glomerulonephritis and tubulointerstitial nephritis to acute kidney injury (AKI) and chronic fibrotic nephropathy (Tecklenborg et al., 2018). The underlying mechanisms linking immune dysfunction to renal injury are diverse, complex and overlapping.

#### 1.2.1 Dysregulation of innate immune responses

Innate immune components, including macrophages, neutrophils, complement, and pattern‐recognition receptors, act as initial responders to infections, toxins, ischemia, metabolic stress, or inflammation. Their activation triggers the recruitment of neutrophils and macrophages, the release of pro-inflammatory cytokines (TNF, IL-1 and IL-6), complement activation, and oxidative stress, leading to endothelial and epithelial injury in both glomeruli and tubules (Marshall et al., 2024; Carlberg et al., 2023). In glomerular disease, deposition of antigen‐antibody complexes induces complement activation and neutrophil recruitment, causing glomerular capillary injury which resembles the type III hypersensitivity mechanism. These immunological imbalances result in mesangial proliferation and changes in glomerular basement membrane (GBM), ultimately leading to sclerosis (Noris and Remuzzi, 2015; Dickinson, 2016).

#### 1.2.2 Adaptive immunity and chronic injury: the outcomes of autoreactivity

Adaptive immunity, including T cells, B cells, and antibodies, plays a vital role in diverse nephropathologies. For example, in IgA nephropathy, dysregulated mucosal IgA and B cells promotes the deposition of an immune complex in the mesangium (Gesualdo et al., 2021; Cheung et al., 2025). Similarly, in lupus nephritis, T cell–mediated injury, pro-inflammatory cytokine release, direct cytotoxicity, and formation of intrarenal tertiary lymphoid structures contribute to renal tissue injury. Persistent and chronic immune activation, therefore, promotes fibrosis through sustained immune cell infiltration, elevated cytokine/chemokine release (e.g., TGF-β from immune and stromal cells), and the connections between immune cells and fibroblasts, which transform acute inflammation into irreversible injury (Jamaly et al., 2021; Zou et al., 2025; Zhao et al., 2025).

#### 1.2.3 The “immune–metabolic–vascular” nexus in kidney disease

Nephropathy commonly occurs in metabolic or vascular conditions such as diabetes, hypertension, and obesity. Immunological pathways intersect with these clinical conditions through persistent low-grade inflammation, also referred to as meta-inflammation, complement dysregulation in diabetic glomerulosclerosis, and context-dependent macrophage polarization within the diabetic kidney. These immune cascades aggravate non‐immune renal injury within renal tissues, leading to long-term adverse outcomes (Khoury et al., 2020; Khoury et al., 2023). Although these immune mechanisms remain inadequately investigated, the contribution of immune-related pathways to metabolic nephropathy is increasingly regarded as a crucial domain of ongoing research to guide timely therapeutic interventions and disease management.

#### 1.2.4 Immune tolerance and abnormal adaptive immunity

Generally, not all immune responses are detrimental, as several immune mechanisms facilitate resolution and tissue repair in affected organs. Regulatory T cells (FoxP3^+^) can reduce inflammation through the secretion of IL-10 and TGF-β, and by inhibitory signaling through CTLA-4 and PD-1, which protect glomerular and tubular structures from injury (Golzari-Sorkheh and Zúñiga-Pflücker, 2023; Ge et al., 2024). M2 macrophages (anti-inflammatory) reduce tissue injury by clearing apoptotic cells and by releasing IL-10, VEGF, and EGF, thereby promoting epithelial regeneration and microvascular repair (Wynn and Vannella, 2016; Yu et al., 2022). Furthermore, certain immune mediators such as resolving, protectins, and maresins halt neutrophil recruitment, thereby leading to enhanced efferocytosis and restoring tissue homeostasis (Ferreira et al., 2022). Additionally, tolerogenic dendritic cells and complement regulators (e.g., factor H) contribute to maintaining immune balance within the renal tissue microenvironment (Petr and Thurman, 2023; Fucikov et al., 2019; Li G. et al., 2025).

However, failure of these immune tolerance mechanisms lead to persistent inflammation and promotes abnormal adaptive tissue repair (Figure 4). Hence, continuous cytokine release, abnormal efferocytosis, tubular epithelial cell senescence, and fibroblast activation enhance fibrosis and promote progression of chronic kidney disease (Yasmeen et al., 2024; Han et al., 2025). Additionally, tubular epithelial cell (TEC) senescence—defined by irreversible cell cycle arrest accompanied by a senescence-associated secretory phenotype (SASP) — has emerged as a key mechanism linking acute immune injury to chronic fibrotic progression. Senescent TECs secrete proinflammatory cytokines (IL-6, IL-8, TGF-β), recruit macrophages, and activate adjacent fibroblasts, creating a self-perpetuating inflammatory microenvironment even in the absence of ongoing active immune insults. This process, termed “inflammaging” in the context of kidney disease, represents a particularly important contributor to CKD progression in aging patients with immune-mediated nephropathies. Therefore, understanding these immune-driven cascades, including enhancing Treg function, modulating macrophage polarization, or supplementing pro-resolving lipid mediators, is crucial and represents valuable therapeutic targets to inhibit irreversible renal damage.

### 1.3 Translational implications and therapeutic potential

Among the diverse immune mechanisms discussed, three interconnected pathways consistently have high translational relevance across multiple nephropathies: B-cell–mediated autoreactivity, complement activation, and fibrosis-related tissue remodeling. These pathways reflect disease heterogeneity and also represent promising targets of current and emerging therapies. Therefore, focusing on these mechanistic axes provides a clinically meaningful framework linking immunopathology with precision therapeutic approaches. Immune‐derived biomarkers, including cytokines, complement activation fragments, and immune cell phenotypes, may provide earlier and more precise prediction of disease progression. Furthermore, they are involved in risk stratification and guide timely intervention for nephropathy earlier than classic markers like proteinuria or eGFR decline (K et al., 2025). For example, in IgA nephropathy, levels of galactose-deficient IgA1 and corresponding anti-glycan autoantibodies strongly associate with disease severity and progression. Other immune biomarkers, such as urinary C5b-9, MCP-1, and IL-18, have been recognized as indicators of inflammatory responses and persistent fibrosis in renal tissues (Berthier and Ju, 2024; Alobaidi, 2025).

Immunomodulatory agents, the novel therapeutic avenue, hold promises for the precise treatment of immunological and autoreactive renal conditions. These drugs, such as B-cell depletion, complement inhibitors, and cytokine blockers, which were initially developed for systemic autoimmune diseases, are now being optimized for renal disease-specific applications (Adytia et al., 2025; McBride et al., 2024). For example, complement inhibitors such as eculizumab and iptacopan have shown promising outcomes in C3 glomerulopathy and atypical hemolytic uremic syndrome. Furthermore, biological drugs such as belimumab (anti-BAFF) and rituximab (anti-CD20) have been shown to be effective in targeting B-cells and can be useful in managing lupus nephritis and IgA nephropathy (Apetrii et al., 2025; van Schai et al., 2022). Similarly, macrophage-modulating compounds and NLRP3 inflammasome inhibitors are widely recognized as emerging and novel therapeutic options for treating diabetic kidney disease and fibrotic nephropathies (Islamuddin and Qin, 2024; Zhan et al., 2023). Therefore, it is inferred that “imbalance between immune system and renal microenvironment is central to disease pathogenesis and represents a promising therapeutic frontier”.

Advances in human molecular genetics and immunogenetics and innovations in DNA/RNA sequencing technologies have shifted traditional nephrology and nephropathy into the era of precision medicine. Genetic variants in immune-system-related genes such as HLA, Fc receptors, and complement components have been associated with susceptibility, clinical phenotype, progression, and treatment response (Bensouna et al., 2025; Kim et al., 2021). Therefore, combining these genetic insights with immune profiling and corresponding transcriptomic data can lead to personalized treatment modalities that stratify individuals most likely to respond to specific immunotherapeutic agents and biologics.

Furthermore, identification of immune triggers is crucial for disease prevention and early intervention. For example, in IgA nephropathy, dysregulation of mucosal immune cascades and disrupted gut microbiota-kidney relationships can predict disease initiation. Similarly, activation of TLRs in renal epithelial and immune cells leads to renal infection and inflammation, thereby driving kidney injury (Cheung et al., 2025; Liu et al., 2023). Therefore, targeting these mechanisms through modulation of mucosal immunity, modulation of the microbiome–kidney interaction, and the use of innate receptor antagonists may prevent the progression of immune-mediated nephropathy.

Notably, it is the immune system that determines whether acute kidney injury (AKI) resolves or advances to chronic kidney disease (CKD), resulting in a concept of an immune trajectory, during which persistent activation of T-cells, macrophages, and fibroblast-immune cell interactions after AKI enhances chronic inflammation and fibrosis (Lee et al., 2024).

The overall discussion throughout this part of the manuscript establishes a conceptual framework between immune dysregulation and nephropathy. The kidney not only serves as a filtration organ but also as an immunologically active site where immunological responses interact with resident immune cells and complement cascades. Loss of immune tolerance, dysregulated cytokine signaling, and persistent complement activation result in tissue injury and progressive fibrosis. Understanding these interconnected mechanisms provides the foundation for mechanism-based disease classification and highlights opportunities for precision immunomodulatory therapies.

## 2 Disease burden and immune-mediated nephropathy and systemic autoimmunity

Immune-mediated renal nephropathies are a group of overlapping conditions which comprise a major constituent of renal pathology and significantly lead to CKD, AKI, and ESRD. These renal conditions are distinguished based on their clinical presentations, pathological characteristics, or underlying immune effector-driven mechanisms involved in disease progression. Clinically, these immune-mediated renal pathologies manifest as nephrotic or nephritic syndrome, glomerulonephritis (GN), or acute renal failure (Cunard and Kelly, 2003). However, each renal condition progressed through distinct immunopathological mechanisms that ultimately result in renal inflammation, structural impairment, and fibrosis.

### 2.1 Lupus nephritis

**FIGURE 1:**
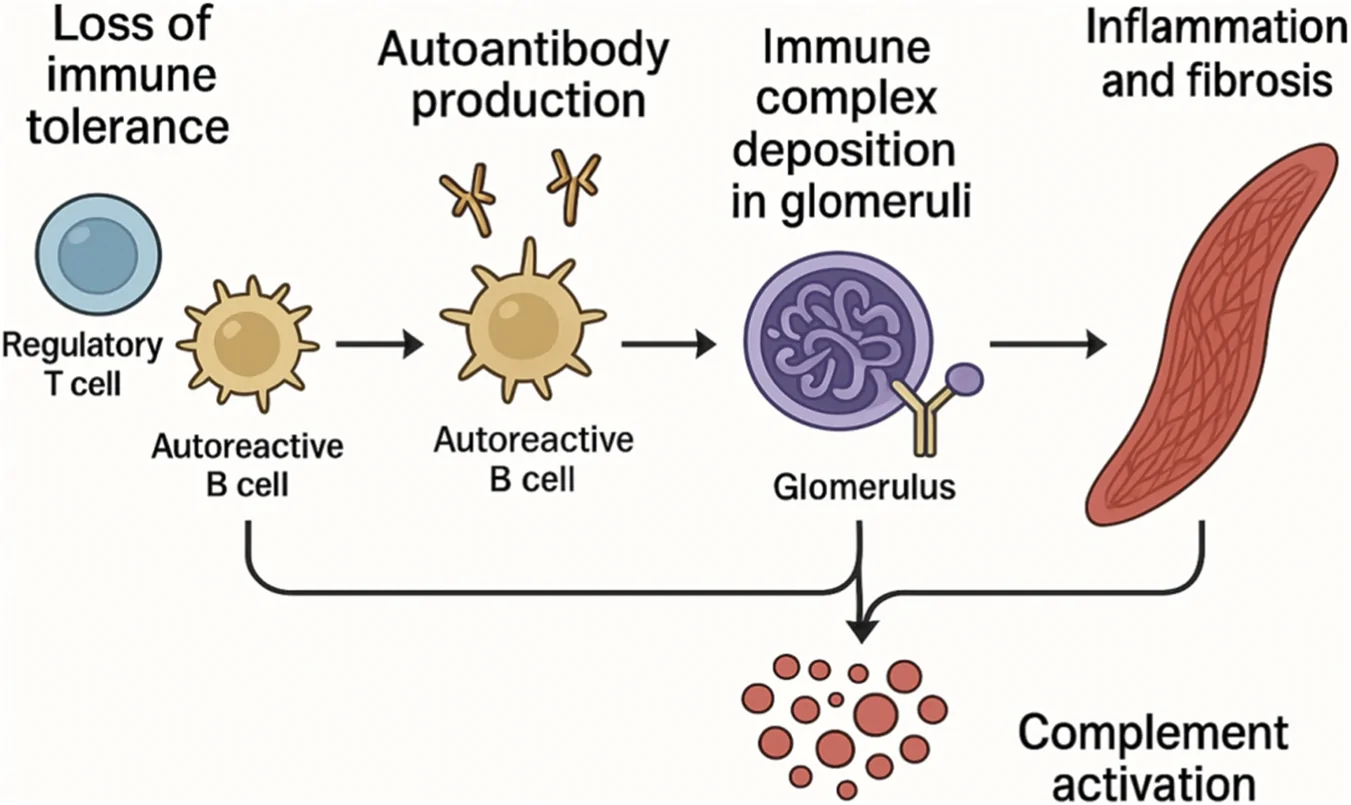
A schematic presentation of lupus nephritis. This conceptual illustration shows the involvement of immune complex deposition and complements activation in glomerular injury.

Lupus nephritis (LN) is a severe outcome of systemic lupus erythematosus (SLE), a complex, autoimmune condition characterized by loss of immune tolerance, autoantibody production, and immune-complex-driven damage (Figure 1). LN usually develops with 3–5 years of SLE onset and progresses to ESRD, especially among women of reproductive and lactating age (Mahajan et al., 2020; Musa et al., 2025).

Early diagnosis is important to prevent irreversible renal damage and depends on monitoring serum creatinine, urine-protein-creatinine ratio, and urinary sediment. In clinical settings, renal biopsy is widely used as a preferred option for disease stratification and therapeutic decision-making; however, non-invasive biomarkers are explored in cases where biopsy is not feasible. Hydroxychloroquine is widely prescribed to SLE patients, whereas moderate-to-severe LN is treated with immunosuppressive medications, including corticosteroids, mycophenolate mofetil, cyclophosphamide, or biologic agents (belimumab and rituximab) (Musa et al., 2025; Mok et al., 2023).

### 2.2 Glomerulonephritis

**FIGURE 2:**
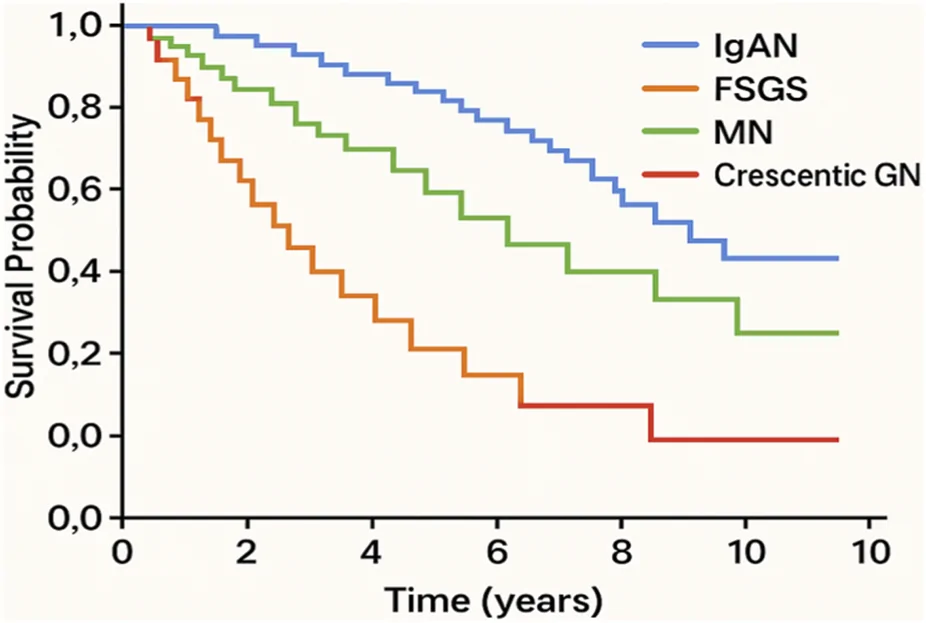
Comparison of survivability among GN subtypes. The figure outlines a comparison of 10-year survivability in 4 GN subtypes. IgA nephropathy (blue): shows the most favorable long-term survival. Membranous nephropathy (green) indicates intermediate survival. FSGS (orange) and Crescentic GN (red) show a significant decline (appear overlapping here), with rapid disease progression and the poorest prognosis. These patients progress rapidly to ESRD, which leads to shorter survival. The figure indicates heterogeneity in disease trajectories and highlights the need for subtype-specific prognostic biomarkers to guide targeted therapeutic interventions and disease management (Abd ElHafeez et al., 2024; Chen et al., 2022; Le et al., 2012; Wang et al., 2023; D’Agati et al., 2013).

Glomerulonephritis (GN) is in a distinct group of immune-mediated renal conditions, characterized by chronic, recurrent inflammation, different therapeutic responses, and progression to ESRD. It differs from metabolic renal disorders, including diabetic nephropathy or hypertensive nephrosclerosis, both in clinical presentation and prognostic outcomes. Renal biopsy is crucial in both identifying GN subtypes and guiding therapeutic decision-making. The European Renal Association registry reported GN, particularly IgAN and focal segmental glomerulosclerosis (FSGS), as one of the leading causes of kidney replacement therapy in Europe (Abd ElHafeez et al., 2024). Furthermore, Crescentic GN (CrGN), also referred to as rapidly progressive GN (RPGN), is a chronic kidney condition that leads to a rapid decline in renal function. During CrGN ‘’crescents’’ structures are formed in the glomeruli and carry the most severe prognosis, with high mortality (Chen et al., 2022). Comparison of 10-year survivability among GN subtypes is illustrated in Figure 2.

### 2.3 Role of non-coding RNAs as biomarkers in detecting IgA nephropathy (IgAN)

**FIGURE 3:**
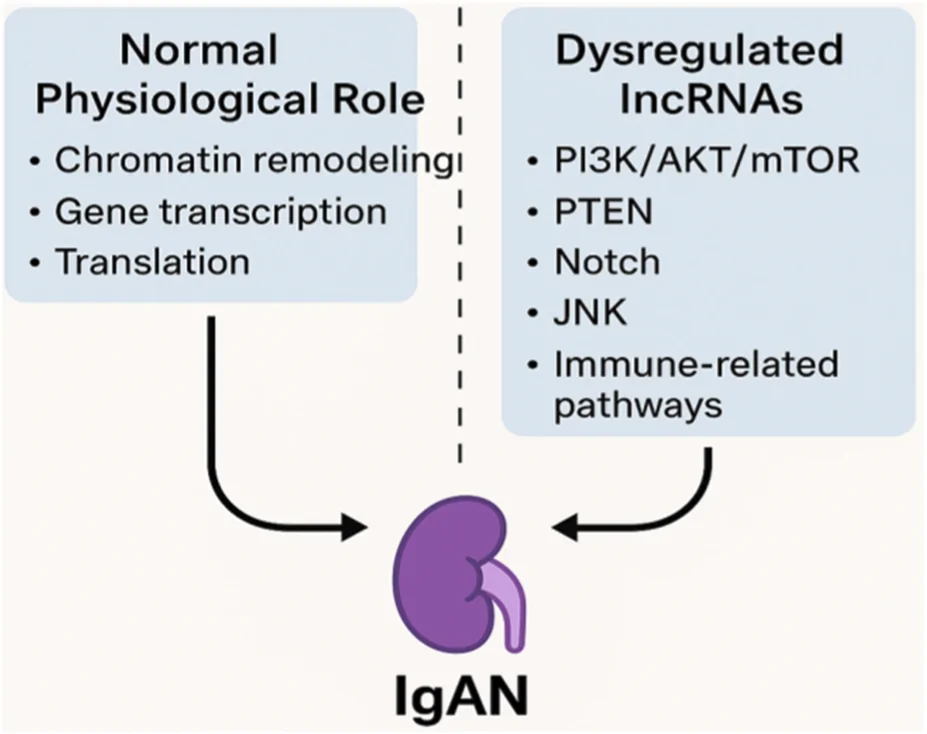
Dysregulation of lncRNAs causes IgAN. Long non-coding RNAs (lncRNAs) play crucial roles in chromatin remodeling, transcriptional regulation, and translational control, thereby maintaining immune homeostasis and renal cellular function. In IgA nephropathy, dysregulated lncRNA expression alters multiple signaling pathways: PI3K/AKT/mTOR, PTEN, Notch, and JNK cascades. These molecular pathways influence immune responses, mesangial proliferation, inflammatory signaling, and extracellular matrix remodeling. These alterations further contribute to immune dysregulation, glomerular injury, and progression of renal pathology, supporting the emerging role of lncRNAs as potential diagnostic biomarkers and therapeutic targets in IgAN.

IgA nephropathy (IgAN) is one of the common forms of glomerulonephritis and is characterized by mesangial deposition of galactose-deficient IgA1, containing immune complexes, with heterogeneous clinical manifestations, ranging from mild hematuria to progressive CKD that ultimately need dialysis or kidney transplantation. Long non-coding RNAs (lncRNAs) have been reported to play a crucial role in modulating IgAN pathogenesis (Figure 3). LncRNAs have been found to modulate several immune responses and renal pathways, including chromatin remodeling, transcription and translation of immune-related genes, and different signaling cascades such as PI3K/AKT/mTOR, PTEN, Notch, and JNK (Almanghadim et al., 2025; Alimohammadi et al., 2025). Changes in the expression levels of lncRNAs have been associated with IgAN, indicating their role in immune dysregulation, mesangial proliferation, and inflammatory responses in renal tissue. Importantly, circulating and exosomal lncRNAs are emerging as promising non-invasive biomarkers, offering potential alternatives to renal biopsy for diagnosis and disease monitoring (Guo et al., 2020; Kademani et al., 2023; Nemazee, 2017). These findings suggest that targeting IncRNA-mediated regulatory networks may provide novel diagnostic and therapeutic opportunities in IgAN. However, significant challenges remain before lncRNAs can serve as routine clinical biomarkers. These include the absence of standardized assay protocols, limited reproducibility across different patient cohorts, uncertainty regarding tissue-specificity of circulating lncRNA signals, and the need for large-scale multicenter validation studies. The potential for non-invasive disease monitoring via exosomal lncRNAs is therefore promising but should currently be regarded as investigational rather than established.

### 2.4 Autoimmunity and renal injury

**FIGURE 4:**
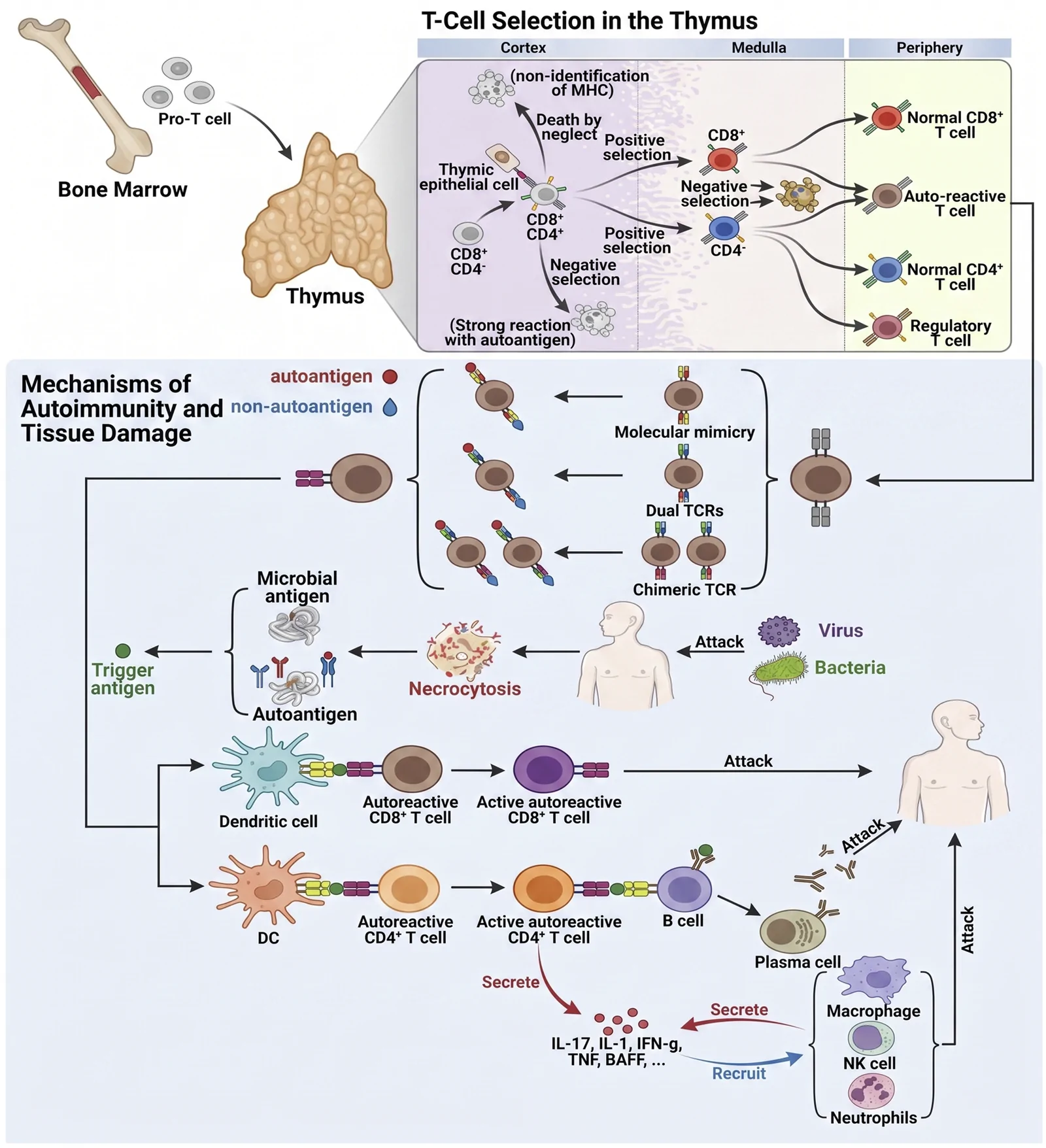
The mechanisms involved in autoreactivity, autoimmunity, and immune tolerance. The figure shows an overview of immune tolerance failure which leads to autoreactive lymphocyte activation, autoantibody production, immune complex formation, and ultimate renal tissue inflammation Conceptually adapted from Tecklenborg et al., 2018; original figure independently redrawn by the authors (Tecklenborg et al., 2018).

Disruption of immune tolerance, both central and peripheral, leads to autoreactivity and autoimmune diseases, where autoreactive B and T cells induce tissue-specific or systemic inflammatory responses (Figure 4). Immune tolerance mechanisms and their roles in autoimmunity are described in Section 1.2. Loss of these mechanisms contribute to autoreactive B and T-cell responses, thereby enhancing renal inflammation. These mechanisms include clonal deletion through cell death (Zheng et al., 2017; Mueller, 2010), induction of anergy mediated by costimulatory pathways such as CTLA-4 (Schwartz, 2003), and generation of regulatory T cells that suppress inflammation (ElTanbouly and Noelle, 2021).

Beyond immune tolerance, effector-molecule-mediated mechanisms also contribute to autoreactivity, resulting in significant kidney injury. During infiltration of autoreactive T cells into target tissues, CD8^+^ T cells kill parenchymal cells and CD4^+^ T cells release cytokines and activate B cells, which in turn promote inflammation. Furthermore, autoantibody-producing B cells form immune complexes, leading to the activation of complement pathways and stimulation of antibody-dependent cellular cytotoxicity (Song et al., 2024; Zhang et al., 2023). Particularly, circulating immune complexes, when deposited in glomeruli, activate the complement system, recruit neutrophils, and induce inflammation, thereby resulting in glomerular injury.

These findings highlight that autoimmune-driven renal injury arises from a convergence of autoreactive immune cells, immune complex deposition, and downstream inflammatory signaling. The integration of these processes provides unifying framework for understanding how systemic immune dysregulation translates into organ-specific damage.

## 3 Renal micro-environment

### 3.1 Kidney as an immunologically active organ

**FIGURE 5:**
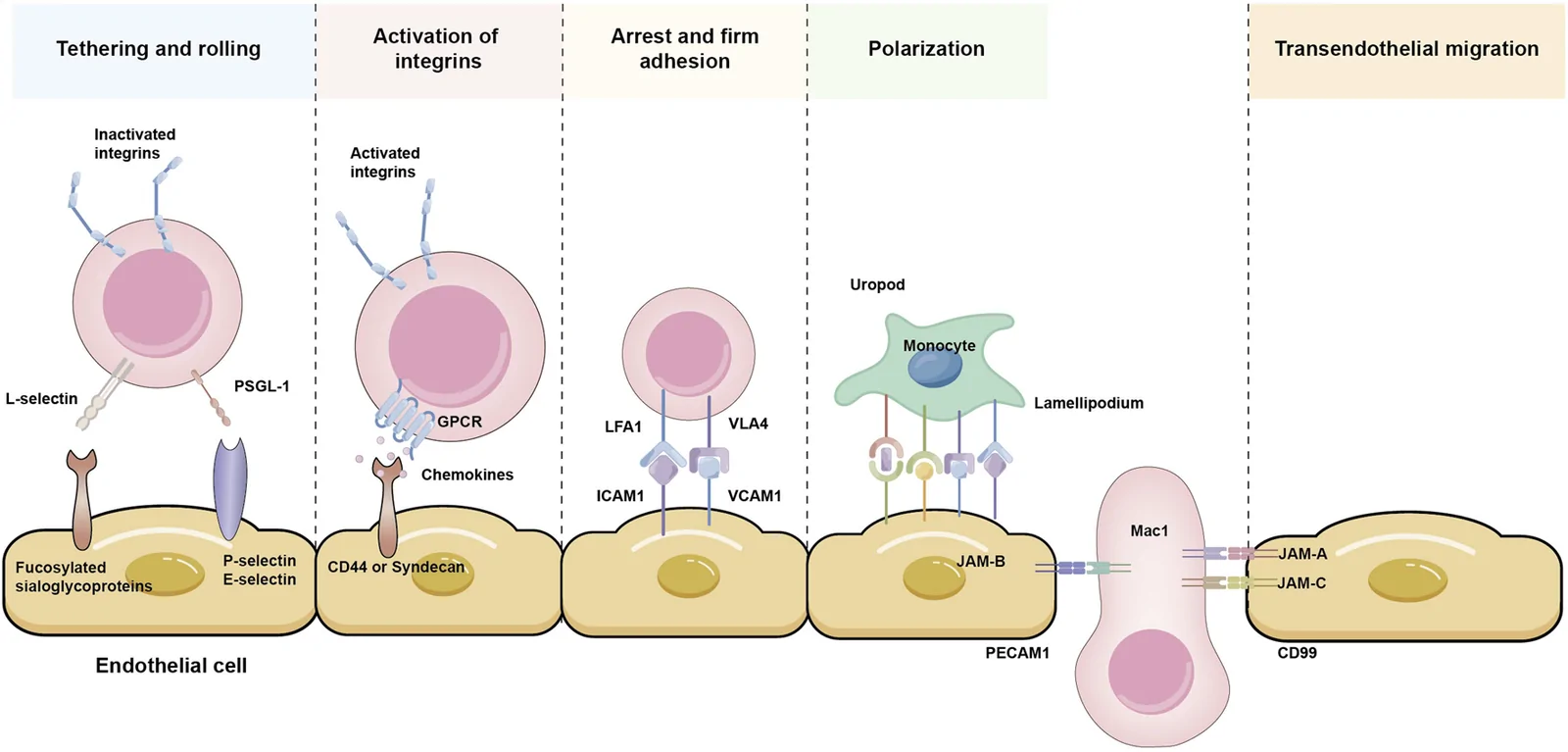
A schematic illustration shows leukocytes recruitment pathways during renal inflammation, indicating the activation of endothelial cells, interaction of adhesion molecules, and migration of immune cell into renal tissue. This process involved various mechanisms. First, selectins mediate the initial tethering and rolling of monocytes along the cytokine-activated endothelial cells. Then, the monocyte interacts with chemokines on the endothelial cells. Activation of GPCR leads to the activation of integrins, and consequent monocyte arrest mediated by the interaction of LFA1 and VLA4 with ICAM1 and VCAM, respectively. Once a monocyte binds to the vascular endothelium, it undergoes polarization, in which chemokine receptors and activated integrins redistribute to the leading edge. After polarization, monocytes migrate toward interendothelial junctions and then transmigrate into the underlying tissues. The members of the JAM family expressed by endothelial cells (JAM-A, JAM-B, JAM-C) interact with the activated integrins on monocytes (LFA1, VLA4, Mac1) and allow the transmigration through tight junctions. Lastly, PECAM-1 (CD31) and CD99 hemophilic engagement and endothelial retraction lead to monocyte extravasation. PSGL: P-selectin glycoprotein ligand-1; GPCR: G-protein-coupled receptors; LFA1: lymphocyte function-associated antigen 1; VLA4: very late antigen 4; ICAM1: intercellular adhesion molecule 1 (ICAM1/CD54); VCAM: vascular cell-adhesion molecule 1; Mac1: macrophage receptor 1; PECAM: platelet/endothelial cell-adhesion molecule 1. (Adopted from: (Medrano-Bosch et al., 2023).

The kidney is a specialized filtration organ that, beyond its physiological functions, serves as an immunologically active site where immune cells and kidney-residual cells interact to shape barrier integrity and immune surveillance (Figure 5). However, under pathological conditions, these crosstalks are dysregulated, resulting in chronic inflammation and progressing to nephropathy. The renal microenvironment, which is a complex network of kidney residual cells and diverse immune cells, including glomerular endothelial cells, infiltrating immune cells, mesangial cells, podocytes, cytokines, extracellular matrix, and tubular epithelial cells (TECs), is a critical determinant of disease progression, significantly contributing to immune-mediated nephropathy.

Renal endothelial cells express adhesion molecules, including ICAM-1, VCAM-1, and E-selectin, facilitating recruitment of leukocytes to the site of injury and contributing to inflammation. TECs and mesangial cells express specific pattern-recognition receptors, inflammatory cytokines, and C3 components. These immune-mediated mechanisms attract immune cells and maintain the inflammatory environment within renal tissue. Similarly, renal parenchymal cells also contribute to persistent immune activation in renal tissue (Tecklenborg et al., 2018; Foresto-Neto et al., 2024; Singh et al., 2023; Molema et al., 2022).

Furthermore, podocytes secrete components of the ECM, which promote the migration and survival of immune cells. These cells express MHC class I and II, costimulatory molecules, and complement receptors, and are directly targeted by antibodies and complement in MN and LN. These mechanisms lead to cytoskeletal rearrangement, fibroblast activation, and fibrosis, which ultimately result in CKD (Medrano-Bosch et al., 2023). The structure of renal microenvironments is summarized in Table 2. Persistent activation of immune effector pathways (such as complement-mediated injury and B-cell–driven autoantibody responses) promotes chronic inflammatory signaling that ultimately support fibrotic remodeling of renal tissue.

**TABLE 2:** Composition of the renal immune micro-environment.

Cell/ component	Physiological role	Pathological role in nephritis	Key mediators/ markers	Therapeutic leverage	References
Endothelium	Barrier, shear sensing	Adhesion, permeability, thrombosis	ICAM-1, VCAM-1, vWF	Anti-adhesion, complement blockade	Cerutti et al. (2017)
Mesangial cells	Matrix support, phagocytosis	Proliferation, matrix expansion	IL-6, C3, PDGF	Anti-cytokine, anti-proliferative	Avraham et al. (2021)
Podocytes	Filtration barrier	Target of antibodies, MAC, apoptosis	PLA2R, THSD7A, NELL1, C5b-9	Anti-CD20, anti-PLA2R guided therapy	Smarz-Widelska et al. (2022)
TECs	Reabsorption, PRR sensing	TLR/NLR activation, fibrosis	TLR2/4, NLRP3, TGF-β	NLRP3, TGF-β modulation	Eun-Kyeong et al. (2015)
Macrophages/ DCs	Tolerance, clearance	M1 skewing, chronic inflammation	CD68, CD163, IL-1β	Macrophage reprogramming	Strizova et al. (2023)
Complement	Immune clearance	Persistent activation, lytic injury	C3d, C5b-9	C5, factor B, C3 inhibitors	Mannes et al. (2021)

### 3.2 Resident and infiltrating leukocytes in renal tissue

Furthermore, resident macrophages and DCs maintain renal tissue integrity and support immune tolerance through IL-10 and TGF-β production and the clearance of apoptotic bodies. However, after injury, macrophage polarization determines whether inflammation resolves or advances to fibrosis. The infiltration of lymphocytes, such as CD4^+^ Th1 and Th17 cells facilitate cytotoxic and inflammatory responses (Niu et al., 2025).

Chronic kidney inflammation leads to the formation of tertiary lymphoid structures (TLS) and has been reported in lupus nephritis, IgA nephropathy, and checkpoint inhibitor–associated nephritis, providing an environment for antigen presentation, B-cell maturation, and epitope spreading (Jamaly et al., 2021; Braunstein, 2025). These structures are commonly accepted as an indicator of refractory disease. Usually, TLS develops as follows: chronic inflammation activates stromal cells; the secretion of chemokines such as CXCL13, CCL19, and CCL21 attracts lymphocytes, B and T cells cluster into distinct renal areas, which is facilitated by follicular DCs, and finally, germinal center-like mechanisms initiate within the kidney. Furthermore, fibroblasts and epithelial cells of the kidney express lymphotoxin-β receptor (LTβR) ligands and act as stromal organizers, a mechanism which is crucial for TLS maturation (Zhao et al., 2024).

### 3.3 Cytokine and chemokine networks

The renal immune system and microenvironment constitute a dense network of cytokines and chemokines which maintain immune tolerance in physiological conditions. However, when this balance is disrupted, infiltration of cytotoxic cells increases, which leads to chronic inflammation and loss of kidney function. For example, IL-6, IL-1β, TNF, IFN-α/β, IFN-γ, BAFF, April, IL-17, IL-23, CCL2, CXCL9, CXCL10, and CXCL13 regulate recruitment of leukocytes, survival of B- and T-cells, polarization of macrophages, and expression of complement system factors. During LN, type I interferon and BAFF signaling contribute to activation of intrarenal B-cell and survival of plasma blast (Lai et al., 2024). Similarly, mucosal cytokines facilitate the production of galactose-deficient IgA1 (Gd-IgA1) during IgAN and TNF and IL-18 recruit neutrophils towards ANCA-mediated activation and NET formation in AAV. These mechanisms can be viable cytokine and chemokine-driven biomarkers for chronic kidney disease detection and promising therapeutic targets in future (Jemelkova et al., 2023; Walulik et al., 2023; Shiratori-Aso and Nakazawa, 2023).

### 3.4 Complement activation and disease progression

Additionally, activation of complement system components further exacerbates tissue injury and thus remains crucial to immune-mediated nephropathy. Specific immune cascades, such as classical pathway activation by immune complexes (e.g., LN, cryoglobulinemic GN, MN), lectin activation by aberrant glycoforms, and alternative pathways, contribute to C3 convertase formation, resulting in the generation of anaphylatoxins C3a and C5a, which support recruitment of leukocytes to the site of injury, thereby causing endothelial damage. Assembly of the terminal C5b-9 membrane attack complex (MAC) induces direct injury to podocytes and other glomerular structures (Gentile and Manenti, 2025). Persistent complement activation facilitates deposition of C3 fragment and C5b-9, which leads to endothelial swelling, direct podocyte injury, and recruitment of C5aR1-expressing myeloid cells. Regulatory proteins, such as CFH, CFI, MCP, THBD, and DGKE, when dysregulated by genetic variants or autoantibodies, can initiate an uncontrolled alternative pathway and result in thrombotic microangiopathy to C3 glomerulopathy (Heidenreich et al., 2024).

Key immunological pathways, including complement activation, macrophage polarization, and inflammasome signaling, collectively shape the inflammatory microenvironment within the kidney. Their interplay determines both the severity of injury/damage and the potential reversibility of disease, emphasizing their importance as therapeutic targets.

## 4 Comparative immunopathogenesis of major immune-mediated nephritis

Immune-mediated nephritis results in diverse systemic and organ-specific conditions, where dysregulated humoral and cellular immunity causes renal injury and functional limitations. These immunological conditions share several molecular events, including inducing antigens, effector pathways, multiple recurrent mechanisms such as loss of self-tolerance, formation of pathogenic autoantibodies or immune complexes, complement activation, recruitment of myeloid and lymphoid effector cells, and a final common pathway of glomerular and tubulointerstitial involvement. This section summarizes key findings in SLE and LN, IgAN, MN, and complement-mediated GN, with emphasis on the immune microenvironment, such as complement system factors, podocyte and mesangial injury, and cross-disease therapeutic innovations.

### 4.1 Immunopathogenesis of systemic lupus erythematosus and lupus nephritis

LN arises from systemic immune dysregulation in SLE, primarily triggered by immune complex-mediated injury and complement activation. The circulating immune complexes, comprising of nucleosomes and anti-dsDNA or anti-nucleosome antibodies, when deposited in glomerular capillary walls and mesangium, may lead to complement activation and leukocyte recruitment, ultimately resulting in proliferative injury (Kościelska-Kasprzak et al., 2014; Obermoser and Pascual, 2010). During such an immune complex–mediated nephritis, tolerance to nuclear antigens is lost, which leads to the production of anti-dsDNA and anti-nucleosome antibodies. Furthermore, dysregulated clearance of apoptotic debris, formation of nucleic acid–containing immune complexes, and engagement of endosomal TLR7/9 in plasmacytoid DCs are implicated as a type I interferon signature (Dai et al., 2025). These interconnected pathways contribute to persistent immune activation, complement-mediated cytotoxicity, and progressive glomerular and tubulointerstitial injury, ultimately leading to chronic renal damage.

### 4.2 Immunopathogenesis of IgA nephropathy (IgAN)

The immunopathogenesis of IgAN is better explained by the “four-hit hypothesis”, which links mucosal dysregulation to renal injury. First, increase in the mucosal immunity dysregulation leads to increased production of galactose-deficient IgA1 (Gd-IgA1). Second, anti-Gd-IgA1 autoantibodies are generated that lead to the formation of circulating immune complexes. Third, the deposition of these complexes in the mesangium, and fourth, activation of NF-κB, MAPK, JAK–STAT, TGF-β and PI3K–Akt signaling, facilitating mesangial proliferation, matrix expansion, and podocyte injury (Yuan et al., 2022). Recent omics studies refine this model and linked Gd-IgA1 biology to genetic variants in glycosylation and mucosal immunity genes (Novak, 2024). The levels of C3, properdin, and C4d within deposits and serum IgA/C3 ratios provide mechanistic biomarkers (Swamy et al., 2025). Together, these mechanisms drive chronic inflammation and progressive renal injury, linking mucosal immune dysregulation with kidney-specific pathological outcomes. Despite its explanatory power, the four-hit hypothesis has notable limitations. It does not fully account for patients with IgAN who lack detectable Gd-IgA1 elevation, nor does it adequately explain why only a subset of individuals with elevated Gd-IgA1 develop nephritis. The relative contribution of mucosal versus systemic immune dysregulation remains incompletely characterized, and the mechanistic basis for variable disease progression—from persistent microscopic hematuria to rapidly progressive renal failure—is not fully captured by this model. These limitations highlight the need for refined disease classification systems that incorporate genetic, proteomic, and microbiome data alongside traditional immunological parameters. Recent clinical trials have substantially advanced the therapeutic landscape for IgAN. The PROTECT trial demonstrated that sparsentan, a dual antagonist of angiotensin type 1 and endothelin type A receptors, significantly reduced proteinuria compared to irbesartan at 9 months, with ongoing evaluation of its long-term effects on eGFR slope (Rovin B, Barratt J, Heerspink H et al. Efficacy and safety of sparsentan versus irbesartan in patients with IgA nephropathy (PROTECT): 2-year results from a randomised, active-controlled, phase 3 trial, The Lancet, 2023; 402, 2077–2090). Additionally, the APPLAUSE-IgAN Phase 2/3 trial showed that iptacopan, a proximal complement factor B inhibitor, significantly reduced proteinuria in IgAN patients with persistent proteinuria on optimized supportive care, providing proof-of-concept that complement inhibition at the alternative pathway level is an effective therapeutic strategy in IgAN (Vlado, Perkovic., Jonathan, Barratt., Brad, Rovin., Naoki, Kashihara., Bart, Maes., Hong, Zhang., Hernán, Trimarchi., Dmitrij, Kollins., Olympia, Papachristofi., Severina, Jacinto-Sanders., Tobias, Merkel., Nicolas, Guerard., Ronny, Renfurm., Thomas, Hach., Dana V, Rizk.(2024). Alternative Complement Pathway Inhibition with Iptacopan in IgA Nephropathy. N Engl J Med, 392(6), 531–543. doi:10.1056/NEJMoa2410316). These findings reinforce the importance of complement dysregulation as a central disease driver and validate the translational relevance of the four-hit framework, while simultaneously pointing to its limitations in capturing the full disease spectrum.

### 4.3 ANCA-associated vasculitis (AAV) and crescentic GN

ANCA-associated vasculitis (AAV) is characterized by the presence of autoantibodies to neutrophil cytoplasmic antigens, myeloperoxidase (MPO) or proteinase 3 (PR3). Usually, little immunoglobulin deposition is observed during biopsies, but activation of the alternative pathway in AAV is now crucial (Poppelaars and Thurman, 2020; Drouzas et al., 2025). Clinically, AAV induces several pathological events in renal tissue, including pauci-immune necrotizing crescentic glomerulonephritis, endothelial damage, fibrinoid necrosis, and monocyte/macrophage and T-cell infiltration. C5a–C5aR1 signaling significantly contributes to AAV immunopathogenesis, and a C5aR1 antagonist, avacopan, has demonstrated efficacy in reducing disease progression and limiting ANCA-mediated injury. The landmark ADVOCATE trial demonstrated that avacopan was non-inferior to prednisone taper at week 26, and achieved superior sustained remission at week 52, with significantly reduced glucocorticoid-related toxicity, supporting C5aR1 blockade as a standard therapeutic option in AAV (Jayne et al., 2021). A critical distinction in AAV lies between PR3-ANCA and MPO-ANCA disease subtypes, which differ in immunopathological mechanism, clinical phenotype, and prognosis. PR3-ANCA is predominantly associated with granulomatosis with polyangiitis (GPA) and carries a significantly higher risk of disease relapse, driven by persistent T-cell memory responses and granulomatous inflammation. In contrast, MPO-ANCA predominates in microscopic polyangiitis (MPA) and is more frequently associated with renal-limited disease, with a comparatively lower relapse rate but greater risk of irreversible renal damage at presentation. These differences have direct implications for therapeutic selection and monitoring strategies. Neutrophil extracellular traps (NETs) play a central mechanistic role in AAV-associated renal injury. ANCA binding to primed neutrophils triggers PAD4-mediated citrullination of histones, facilitating NET extrusion. These NETs expose and release MPO and PR3, which further activate complement—particularly through the alternative pathway—and damage glomerular endothelial cells. In lupus nephritis, a parallel mechanism operates whereby NET-derived nucleic acids stimulate plasmacytoid dendritic cells via TLR7/9, sustaining type I interferon production. The shared NET-complement axis across AAV and LN represents a compelling cross-disease therapeutic target, with ongoing investigation of PAD4 inhibitors and NET-clearing strategies.

### 4.4 Complement-mediated glomerulopathies (C3G, aHUS, IC-MPGN)

Complement dysregulation is a key immunological event that occurs in C3 glomerulopathy (C3G), which is atypical hemolytic uremic syndrome (aHUS), and a subset of membranoproliferative glomerulonephritis (MPGN). C3G is characterized by C3 deposition with minimal immunoglobulin and includes C3 glomerulonephritis and dense deposit condition (Pickering et al., 2013; Smith et al., 2019). Studies have revealed that genetic mutations and autoantibodies against different factors, including factor H, factor I, C3, factor B, or C5, contribute to glomerular injury (Heidenreich et al., 2024). These events explain pure complement-driven immunopathology of nephropathy: dense deposit disease and C3GN with persistent activation and deposition of C3, and aHUS with endothelial injury and thrombotic microangiopathy. Furthermore, complement inhibition through eculizumab, ravulizumab, iptacopan, and factor D/B blockers have been found effective in inhibiting these pathways, thereby protecting against organ injury during C3G conditions (Apetrii et al., 2025).

Although individual nephropathies exhibit distinct and molecular features, they share common immunological drivers, changes in the dominance and interaction of these pathways contribute to disease heterogeneity while maintaining a core framework of immune-mediated injury.

### 4.5 Immunopathogenesis of primary membranous nephropathy (MN)

Primary MN is an antigen-specific, podocyte-targeted autoimmune renal condition in adults. Clinically, it is presented with proteinuria, hypoalbuminemia, and hyperlipidemia, characterized by thickened glomerular capillary walls. There is evidence of spontaneous remission of MN in about one-third of the patients, whereas experimentally 40% cases have been reported to reach ESDR (McGrogan et al., 2011; Valentini, 2016). Podocyte antigens, such as M-type phospholipase A2 receptor (anti-PLA2R1 antibodies), THSD7A, EXT1/2, NELL1, Sema3B, NCAM1, PCDH7, HTRA1, NTNG1, recognized MN an antigen-defined autoantibody disease or organ specific autoimmune conditions, which was previously considered as an idiopathic entity (Beck et al., 2009; Couser, 2017; Ronco and Debiec, 2007). Circulating anti-PLA2R1 IgG titers and glomerular PLA2R staining are now used to assess disease activity and recognized as sensitive and specific biomarkers of MN to detect and monitor disease severity (Efe et al., 2024; Murtas et al., 2024).

Overall, the mechanistic hallmarks in MN include the presence of anti-podocyte antigen autoantibodies, deposition of immune complexes along the GBM, and activation of the complement system, often via lectin and alternative pathways. These molecular mechanisms cause sublethal podocyte injury and proteinuria, thereby exacerbating disease progression.

### 4.6 Immune checkpoint inhibitors (ICI) and nephritis

Immune checkpoint inhibitors (ICI) are innovative treatments for autoimmune conditions and cancers. These molecules are designed to disrupt peripheral tolerance, thereby enhancing anti-tumor immunity. However, ICI can trigger several immune-related adverse events, which lead to renal inflammation, such as acute interstitial nephritis (AIN) and GN. The immunopathological events that occur during ICI-induced nephritis include interstitial infiltration of T cells, often with eosinophils, tubulitis and variable glomerular lesions, and frequent co-exposure to PPIs or other nephritogenic drugs (Tocheva and Mor, 2017; Sun et al., 2025; Younis and Gribben, 2024). Furthermore, inhibition of PD-1/CTLA-4 signaling may also trigger the production of autoreactive T-cells, enhance their reactivity against renal antigens, and facilitate TLS-like structures, thereby increasing the risk of nephritis (Zhou et al., 2024). Therefore, while working with onco-immunology for controlling cancer, the systemic modulation of checkpoints should be considered to prevent renal autoimmunity, thereby inhibiting the onset of chronic kidney disease.

## 5 Cross-disease cascades and shared nephritic pathways

### 5.1 Autoreactivity, autoantibody class, and effector functions

Autoantibodies produced during autoimmune conditions drive pathological events in the body. These pathologies are governed by their distinct properties, such as antigen specificity (nuclear, MPO/PR3, Gd-IgA1, PLA2R, NELL1), the isotype and subclass (e.g., IgG1/3 with strong complement activation vs. IgG4-predominant profiles), and glycosylation, affinity maturation, and capacity to form large, circulating complexes (Ludwig et al., 2017). However, nephropathy is a combination of several immunological events as follows: LN and cryoglobulinemia are triggered by deposition of immune complexes; direct activation of primed neutrophils induces AAV; MN is caused by *in-situ* binding to podocyte antigens; IgAN is driven by mesangial Gd-IgA1 complexes. Thus, the combination of antigen, antibody quality, complement and immune system involvement can explain the patterns and severity of renal injury and is suggested to be a viable marker for disease prediction and disease management (Roccatello et al., 2018; Hu et al., 2011).

### 5.2 Complement pathways as unifying modules

Complement activation (see Section 3,4) significantly participates immunological responses during nephropathy. These mechanisms are governed as follows: classical pathways are involved in IC-mediated LN and MN; the lectin pathway contributes to IgAN and infections; and alternative pathways work as the final modulators in exacerbating pathogenesis in IgAN, AAV, C3G, aHUS, and diabetic nephropathy. Given the shared mechanistic patients may be classified into high complement endotypes, which are then selected for pathway inhibition (Vivarelli et al., 2024).

### 5.3 Innate drivers in nephropathy: macrophages, NETs, and inflammasomes

Innate immune drivers play a crucial role in persistent nephropathies across all immune-mediated nephritis. After recruitment into glomeruli and tubulointerstitium, macrophages are differentiated into proinflammatory M1 subtypes that release proinflammatory cytokines, increasing endothelial and epithelial injury. Normally, the M1 phenotype is polarized into M2; however, when this transition fails, it causes a persistent inflammatory response, a common event observed in lupus nephritis, IgA nephropathy, ANCA-associated vasculitis (AAV), and C3 glomerulopathy. Similarly, ANCAs induce excessive NET formation in AAV and LN, triggering multiple immunological responses: exposing proteases, histones, and myeloperoxidase (these responses damage glomerular capillaries and activate neutrophils), and in LN, it facilitates deposition of immune complexes and promotes DCs to support autoimmunity (Zhu et al., 2025; Ning et al., 2024). NLRP3 inflammasome is another driver of inflammation, which is activated in TECs and podocytes and leads to pyroptotic injury (Yamashita and Kramann, 2024; Meng et al., 2025). Immunometabolic reprogramming further shapes innate immune cell behavior in nephropathies. Proinflammatory M1 macrophages rely predominantly on aerobic glycolysis and the pentose phosphate pathway, while anti-inflammatory M2 macrophages favor oxidative phosphorylation and fatty acid oxidation. Persistent glycolytic metabolism in glomerular and interstitial macrophages sustains NF-κB and HIF-1α activation, perpetuating inflammation. Emerging evidence suggests that targeting metabolic checkpoints—such as mTORC1 inhibition to enforce M2 polarization or itaconate supplementation to suppress NLRP3 activation—may offer novel immunometabolic therapeutic avenues in immune-mediated nephropathies.

### 5.4 Fibrosis: a cross-disease shared endpoint

Despite several initiating events, immune-mediated nephritis concludes with progressive fibrosis. These events include tubular atrophy, capillary rarefaction, accumulation of extracellular matrix, and activation of myofibroblasts, leading to persistent immune activations which inhibit epithelial repair cascades. Single-cell analyses demonstrate that chronic immune signaling drives profibrotic stromal cell states (Huang et al., 2023).

Single-cell RNA-sequencing and transcriptomic analyses reported distinct fibroblast subtypes which contribute to LN, IgA nephropathy, AAC, and MN. These fibroblast subpopulations include “inflammatory fibroblasts” expressing CCL2 and CXCL12, “matrix-producing fibroblasts” expressing COL1A1, COL3A1, and FN1, and a disease-agnostic population “kidney myofibroblast lineage cells” associated with ACTA2 and TAGLN. Interaction between infiltrating macrophages, injured tubular cells, and these fibroblast subpopulations further increases inflammatory responses in renal tissue (Chambers et al., 2019; Barron et al., 2024). Across these cross-disease immune modules, a hierarchical signaling architecture can be delineated. Innate pattern-recognition receptors—TLR–MyD88 and cGAS–STING—function as upstream sensors of danger signals, including self-nucleic acids, microbial products, and complement opsonins. These converge on NF-κB and JAK–STAT as central transduction hubs, amplifying proinflammatory cytokine production, immune cell recruitment, and autoantibody class-switching. Downstream, TGF-β/Smad signaling operates as the principal executor of fibrotic remodeling, translating chronic immune activation into irreversible structural injury. Importantly, this hierarchy is not uniform across diseases: cGAS–STING is predominantly activated in LN through self-nucleic acid sensing from apoptotic debris, while NLRP3 inflammasome priming is more prominent in IgAN, AAV, and diabetic nephropathy. JAK–STAT activation has received the strongest clinical validation in LN, with evidence from baricitinib and voclosporin trials. In contrast, NF-κB inhibition remains an active but primarily preclinical area across nephropathies. Recognizing this hierarchy enables more rational therapeutic targeting—inhibiting the appropriate upstream sensor or central transducer rather than broadly suppressing downstream effectors.

## 6 Biomarkers and monitoring of nephropathy: from validated clinical tools to exploratory molecular indicators

Biomarkers discussed in this review do not carry the same degree of clinical readiness. Some of these biomarkers are already utilized in routine nephrology practice or guideline-based protocols, whereas others remain emerging adjuncts or exploratory molecular candidates. Biomarkers associated with B-cell activation and complement signaling are among the most clinically advanced indicators currently used for disease classification and treatment stratification. The section below differentiates validated clinical tools from those that are promising mechanistically but still require assay standardization, multicenter validation, and estimation of significant value over traditional clinical, serologic, and histopathologic evaluation.

### 6.1 Validated serologic and pathology-based clinical tools

Multiple key serological markers have been identified across immune-mediated nephropathies, enabling earlier identification, risk stratification, and therapeutic decision making. These serological markers are not equivalent in clinical maturity. Some of these biomarkers, such as ANA, anti-dsDNA, anti-C1q, complement C3/C4, MPO-/PR3-ANCA, serum anti-PLA2R are established or guideline-supported tools in routine clinical practice, whereas other anti-THSD7A, anti-NELL1, Gd-IgA1, anti-Gd-IgA1, IgA/C3 ratio, factor H/I antibodies, C3, and sC5b-9 currently remain as adjunctive indicators. Recently, KDIGO-2024 and KDIGO-2025 have incorporated these serological panels into disease identification and treatment algorithms (Disease and Work, 2024).

### 6.2 Emerging adjunctive biomarkers

Uritary cytokines, chemokines, and cell-based markers are promising non-invasive adjuncts for assessing disease activity; however, most remain exploratory and have not yet achieved analytical standardization or multicenter validations required for adoption in routine clinical practice. Adjuncts urinary biomarkers include MCP-1, NGAL, CXCL10, TWEAK, VCAM-1, soluble CD163, and exosomal signatures. These markers are significantly associated with disease activity, reflecting renal inflammation, tubular damage, and macrophage activation. These are also helpful in investigating other renal complications with similar clinical outcomes, thereby minimizing repeated kidney biopsies and improving overall diagnosis and therapeutic decision-making (Vrabie et al., 2025).

### 6.3 Exploratory urinary, exosomal and transcriptomics markers

Standardized histologic activity, and chronicity indices, immunofluorescence patterns, and electron microscopy are validated key tools for evaluating immune-mediated nephropathies. In contrast, antigen-specific staining panels such as PLA2R, THSD7A, NELL1, and exostosins, complement split products, such as C3d and C5b-9 and advanced transcriptomic panels which are recommended for clinical management but remain uneven across diseases and practice settings. The panels are particularly crucial for differentiating primary MN from secondary, confirming C3G versus immune-mediated MPGN, and optimizing the classification and risk stratification of high-risk LN classes (Choi et al., 2023; Bajcsi et al., 2023).

### 6.4 Proposed clinical workflow

A biomarker workflow can be established for immune-mediated renal conditions, moving from broad screening to disease-specific identification and disease monitoring. The screening can be started from serological characterizations, including evaluating antinuclear antibodies, ANCA, complement complex levels such as C4 and C5, and disease-determining autoantibodies like anti-PLA2R MN or galactose-deficient IgA1 (Gd-IgA1) for suspected IgA nephropathy (Kant et al., 2022; Laigle et al., 2023). Serological assessment is followed by biopsy with standardized immunofluorescence and electron microscopy, along with antigen panels. For cases with suspected CMG/aHUS, specific complement profiling is recommended, such as quantification of C3/C4, factor H and I, C3 nephritic factor, anti–factor H antibodies, and soluble C5b-9.

In clinical workflow, validated biomarker should be used for diagnosis and management, whereas investigational predictors as are applied as complementary research-phase tools until broader validation are standardization are achieved. For known cases, longitudinal assessment of autoantibody levels such as anti-dsDNA, anti-PLA2R, ANCA, along with urinary biomarkers (for example, proteinuria, and where available, MCP-1, NGAL, CXCL10 or other exploratory markers) can be used for relapse prediction, response assessment, and treatment planning, reducing the need for repeated biopsy and enabling biomarker-guided care for disease management (Ronco et al., 2021; Harms et al., 2023). Commonly used in identification and risk assessment across various renal pathologies are summarized in Table 3.

**TABLE 3:** A list of biomarkers used in immune-mediated nephropathy: their role in diagnosis and prognosis.

Disease/ Condition	Biomarker	Source/ assay or diagnostic approach used	Main role (diagnostic/ prognostic/ predictive)	Clinical readiness	References
Lupus nephritis (LN)	Anti–double stranded DNA (anti-dsDNA) antibodies	Serum ELISA/ Farr assay	Diagnostic criterion for SLE, correlates with renal activity, useful for flare prediction when combined with complement levels	Validated (guideline-endorsed)	Linnik et al. (2005)
	Anti-C1q antibodies	Serum ELISA	Associated with proliferative LN, predicts renal flares and poor outcomes more strongly than anti-dsDNA in some cohorts	Validated (guideline-endorsed)	Yang et al. (2012)
	Anti-nucleosome antibodies	Serum ELISA	Early marker of SLE, linked to nephritis and may precede anti-dsDNA positivity; aids risk stratification in seronegative patients	Validated (guideline-endorsed)	Abdel Gawad et al. (2014)
	Complement C3/ C4, CH50	Serum immunonephelometry	Low C3 and C4 reflect immune complex driven activation, associated with active nephritis and risk of ESRD; persistent isolated C3 hypocomplementemia is strongly prognostic	Validated (guideline-endorsed)	Andrulli et al. (2025)
	Urinary MCP-1, NGAL, TWEAK	Urine ELISA/ multiplex	Reflect intrarenal inflammation and tubular injury; improve discrimination between active and chronic lesions and predict treatment response beyond proteinuria	Adjunctive/ Emerging	Abdel Gawad et al. (2014)
​	Glomerular C3, C1q, C4d, C5b-9	Renal biopsy IF/ IHC	Patterns and intensity of complement deposition correlate with histologic class and chronicity index; C5b-9 predicts progression and is a potential theragnostic marker for complement blockade	​	Abdel Gawad et al. (2014)
IgA nephropathy (IgAN)	Galactose deficient IgA1 (Gd-IgA1)	Serum ELISA/ lectin assay	Pathogenic core of the four-hit model; elevated levels in many patients and some relatives; candidate diagnostic and risk biomarker	Adjunctive/ Emerging	Elíasdóttir et al. (2023)
	Anti-Gd-IgA1 autoantibodies	Serum ELISA	Form circulating immune complexes, associated with more aggressive disease and proteinuria; useful for prognostic enrichment in trials	Adjunctive/ Emerging	Sharma et al. (2025)
	Complement deposition (C3, C4d, MBL, FHR5)	Renal biopsy IF/ IHC	Mesangial and capillary wall C3 and C4d, and FHR5 deposition indicate lectin and alternative pathway activation and predict faster decline in eGFR	​	Ștefan et al. (2023)
	Serum C3; C3/C4 ratio	Serum immunonephelometry	Low C3 and reduced C3/C4 ratio associate with more severe histologic lesions and progression to ESKD	​	Ștefan et al. (2023)
	Exosomal lncRNAs (for example, lncRNA-G21551, ICAM-1-related lncRNAs)	Plasma or urinary exosomes, RNA-seq/ qPCR	Non-invasive markers of disease presence and fibrosis; ICAM-1-related lncRNA expression predicts progression and tubular injury	Exploratory/ Investigational	Li et al. (2025)
	lncRNA CRNDE	Renal macrophages, expression profiling	Drives NLRP3 inflammasome activation and promotes inflammatory progression; potential predictor of aggressive, macrophage rich IgAN	Exploratory/ Investigational	Shen et al. (2022)
	lncRNA FGD5-AS1	Peripheral blood and renal cells	Upregulation dampens PTEN-mediated JNK/c-Jun signalling and is associated with milder disease in children; candidate protective biomarker	Exploratory/ Investigational	Sun et al. (2023)
Primary membranous nephropathy (PMN)	Anti-PLA2R antibodies	Serum ELISA/ immunofluorescence	Highly specific diagnostic marker for PMN; baseline titer correlates with risk of progression and decline in titre precedes clinical remission (predictive marker)	Validated (guideline-endorsed)	Ragy et al. (2023)
	Glomerular PLA2R antigen	Renal biopsy IF	Confirms PLA2R-associated PMN, helps distinguish primary from secondary MN particularly when antibodies are low or treated	Validated (guideline-endorsed)	Lu et al. (2024)
	Anti-THSD7A antibodies	Serum ELISA/ IF	Identify a distinct subset of MN, often with higher malignancy association; useful for cancer screening and relapse monitoring	​	Xu et al. (2024)
	IgG subclass pattern (IgG4-dominant)	Renal biopsy IF for IgG subclasses	IgG4-dominant deposits support primary MN; IgG1/2/3 rich patterns suggest secondary MN and influence search for systemic disease	​	Kattah et al. (2016)
	Glomerular C3, C4d, C5b-9; serum sC5b-9	IF/ IHC; serum assay	Reflect complement activation, particularly lectin and alternative pathways; intensity of C5b-9 correlates with podocyte injury and proteinuria	​	Kotsalas et al. (2025)
C3 glomerulopathy (C3G)	Serum C3	Serum immunonephelometry	Markedly reduced C3 with near normal C4 suggests alternative pathway dysregulation; low C3 is part of diagnostic work-up and prognostic for relapse	​	Ricker et al. (1991)
	C3 nephritic factor (C3NeF) and C5 nephritic factors	Serum functional assays	Autoantibodies that stabilize C3/C5 convertases; define pathogenic subtype and may stratify patients for proximal complement inhibitors	​	Hauer et al. (2023)
	Complement gene variants (CFH, CFI, C3, CFB, CFHRs)	Germline sequencing	Identify inherited drivers of complement dysregulation, inform recurrence risk after transplant and choice of targeted therapy	​	Meuleman et al. (2023)
	Glomerular C3 with scant or absent Ig	Renal biopsy IF	Diagnostic hallmark distinguishing C3G from immune complex mediated MPGN; chronicity indices predict long term outcome	​	Kirpalani et al. (2020)
ANCA-associated vasculitis (AAV)	PR3-ANCA and MPO-ANCA titers	Serum immunofluorescence and ELISA	Central diagnostic markers; PR3-ANCA associates with higher relapse risk, MPO-ANCA with more renal limited disease	Validated (guideline-endorsed)	Can et al. (2026)
	Serum C3; markers of alternative pathway activation (C3a, C5a, sC5b-9)	Serum assays	Low C3 and elevated C5a or sC5b-9 associated with severe renal involvement, thrombotic microangiopathy and poorer kidney survival	​	Kesarwani et al. (2024)
	Urinary MCP-1, monocyte and neutrophil gene signatures	Urine biomarkers, RNA profiling	Reflect ongoing intrarenal vasculitis and predict response to avacopan or rituximab-based regimens	Adjunctive/ Emerging	Song et al. (2024)
Complement mediated TMA/ aHUS	Pathogenic variants in CFH, CFI, C3, CFB, CD46, CFHRs; anti-factor H antibodies	Germline sequencing; serum autoantibody assays	Define complement mediated TMA, guide long term prognosis, transplant risk, and need for prolonged C5 blockade	​	Afshar-Kharghan (2025)
	Serum sC5b-9, C3, C4	Serum assays	Indicate terminal pathway activation and help monitor pharmacodynamic response to eculizumab or ravulizumab	​	Jodele et al. (2025)
Cross-disease (LN, IgAN, MN, C3G, AAV) Cross-disease	Urinary or plasma sC5b-9, C3a, C5a	Body fluids	Shared indicators of complement overactivation that correlate with activity and may act as pharmacodynamic markers in trials of complement inhibitors	​	Budkowska et al. (2023)
	Histological chronicity scores (IFTA, glomerulosclerosis, vascular lesions) combined with immune markers	Renal biopsy scoring	Integrate immune and structural damage; strong predictors of ESKD across immune mediated nephritides and useful as composite endpoints	​	Leatherwood et al. (2019)

## 7 Comparative outcomes of immune-mediated nephropathies

Like other chronic diseases, such as tumors and cancers, relapses and recurrence are the major health concerns across immune-mediated nephropathy. In LN, relapse appears in approximately 30%–50% of cases. It usually occurs in that persistent serologic activity, such as higher anti-dsDNA titers or low complement levels, suboptimal adherence to medications, or inadequate maintenance interventions (Almaani et al., 2017; Mohan et al., 2023). However, AAV appears in a distinct pattern, where relapse risk is strongly affected by ANCA type, with PR3-ANCA-related disease being more common than MPO-ANCA cases, by tapering approaches or complement-cascade activation. In MN, serologic relapses, such as increasing anti-PLA2R levels, commonly appear as a clinical relapse and are considered the early warning sign of disease severity (De Vriese et al., 2017). Furthermore, relapse in IgAN advanced as progressive decline even in individuals with clinically stable conditions (Shen et al., 2022).

Treatment-related toxicity of these immune-mediated conditions varies across drug types. Alkylating agents and long-term high-dose corticosteroids can cause infertility, malignancy, metabolic complications, and serious infection. B-cell–depleting therapies may lead to hypogammaglobulinemia and alter response to vaccination, whereas complement inhibitors increase vulnerability to encapsulated bacteria, demanding vaccination programs and antimicrobial prophylaxis (Rondeau et al., 2020). Therefore, defining immunologic endotypes across LN, AAV, MN, and IgAN, including autoantibody signatures, complement activation markers, and cellular profiles assessment, is useful to tailor immunosuppression more precisely, which helps reduce treatment-related toxicity (Izcovich, 2024; Rached et al., 2024; Zhang et al., 2025).

Furthermore, there are system-level and access differences across these renal conditions. For example, access to renal biopsy, availability of antigen-specific assays such as PLA2R, NELL1 or exostosins, access to complement profiling, and availability of biologic therapies remain uneven across different areas of the world. Similarly, in several low-income and middle-income settings, suboptimal diagnostic facilities result in delayed early diagnosis of high-risk cases and reliance on empiric steroid-heavy therapies that worsen long-term renal and systemic outcomes. These variabilities in resource utilization and access highlight the need for simpler, cost-effective disease diagnosis tools in such low-income settings (Thurlow et al., 2021).

## 8 Future perspectives and research directions

Future research and management directions in immune-mediated nephropathy increasingly focus on mechanism-guided tailored care, which combines molecular profiling with clinical assessment for better decision-making. Advanced multi-omics technologies, including single-cell RNA sequencing, emerging transcriptomics, proteomics, metabolomics, and high-resolution complement and autoantibody profiling, are being applied to develop “immune-kidney atlases.” These technologies can resolve intrarenal immune cell population, endothelial, and stromal niches, and can identify dynamic signaling networks that vary across different renal conditions such as LN, IgA, MN, AAV, and C3G. By assessing these molecular signatures along with advanced histology, serology, and patient-centered outcomes, these approaches enable the identification of dynamic networks, optimize risk stratification, and improve predictive models that move beyond traditional approaches to real-time diagnosis and early interventions.

At the therapeutic level, next-generation approaches are likely to shift from blunt immunosuppression to tolerance-inducing and tailored therapeutic interventions. These next-generation strategies include low-dose IL-2 and methods to apply cell-based Treg therapies, antigen-specific tolerogenic vaccines developed against nephrogenic targets such as PLA2R, MPO/PR3, Gd-IgA1 constructs, engineered cell approaches such as CAR-Treg or modified B-cells manipulated to delete or neutralize pathogenic clones. Although these approaches are still experimental and under development, they closely align with the mechanistic insights discussed and reviewed in this manuscript and direct the researchers toward efficient disease management with less overall adverse effects and toxicity.

Furthermore, clinical trial designs need to be advanced and mechanism-tailored for better outcomes. Future trials need to stratify patients using novel serological, histological, and molecular markers, which include PLA2R titers, complement signatures and activation profiles, interferon scores, Gd-IgA1, or other relevant immune-complex profiling. Adaptive designs and composite endpoints should be adopted that integrate proteinuria, estimated glomerular filtration rate slope (eGFR slope), histological parameters, chronicity indicators, and detectors of molecular remission to capture meaningful outcomes. Furthermore, embedded biospecimen collections and multi-omics approaches should be applied to optimize these endotypes and to differentiate actual mechanistic responders from non-responders or partial responders.

Future therapeutic strategies are increasingly prioritizing modulation of B-cell activity, complement cascade regulation, and prevention of fibrosis progression. Additionally, investigators should focus on patient-reported clinical and phenotypic outcomes and long-term survivability. As immune-mediated renal conditions significantly affect fatigue, cognition and emotional status, fertility and pregnancy-related outcomes, employment and daily activities, and mental wellbeing and physical activities, which are usually ignored or misdiagnosed, while assessing biomarkers such as creatinine levels, proteinuria, or biopsy-related indicators. Therefore, combining validated patient-reported measures and data alongside innovative molecular and clinical indicators would be crucial to guiding therapeutic regimens tailored to disease features that will not be effective only in managing inflammation and maintaining the kidney normal physiological status, but will also improve the adherence and overall long-term survival of the affected individuals.

## 9 Conclusion

Immune-mediated nephropathies arise from interconnected immunological mechanisms, including autoantibody- and complement-mediated damage, immune complex deposition, and dysregulated tissue remodeling, which collectively shape diverse clinical and histopathological outcomes. While these mechanisms operate in varying combinations across diseases such as lupus nephritis, ANCA-associated vasculitis, IgA nephropathy, membranous nephropathy, and complement mediated glomerulopathies, they converge on pathways that determine diseases progression and reversibility.

Translating this mechanistic understanding into clinical practice is essential. Integration of serological and tissue-based biomarkers, complement profiling, and immunogenetic insights can enable more precise diseases classification, early diagnosis and risk stratification. These advances support a shift toward mechanism-based and personalized therapeutic strategies, including targeted biologics, B-cells-directed therapies, and complement inhibitors, reducing reliance on non-specific immunosuppression. Future progress will depend on linking multi-omics data longitudinal clinical outcomes and incorporating these insights into adaptive clinical trial designs and routine care. Such approaches have the potential to improve renal outcomes, minimized treatment-related toxicity, delay progression to end-stage renal disease, and enhance patient quality of life across diverse healthcare settings.

## Glossary

AAV: ANCA-associated vasculitis
AIN: Acute interstitial nephritis
AKI: Acute kidney injury
ANA: Antinuclear antibody
ANCA: Antineutrophil cytoplasmic antibody
APRIL: A proliferation-inducing ligand
BAFF: B-cell activating factor
C3G: C3 glomerulopathy
cGAS: Cyclic GMP-AMP synthase
CKD: Chronic kidney disease
CMG: Complement-mediated glomerulopathy
ECM: Extracellular matrix
eGFR: Estimated glomerular filtration rate
ESRD: End-stage renal disease
GBM: Glomerular basement membrane
Gd-IgA1: Galactose-deficient IgA1
GN: Glomerulonephritis
HIF-1α: Hypoxia-inducible factor-1α
ICI: Immune checkpoint inhibitor
IgAN: IgA nephropathy
LN: Lupus nephritis
lncRNA: Long non-coding RNA
MAC: Membrane attack complex
MCP-1: Monocyte chemoattractant protein-1
MN: Membranous nephropathy
MPO: Myeloperoxidase
NET: Neutrophil extracellular trap
NGAL: Neutrophil gelatinase-associated lipocalin
NLRP3: NLR family pyrin domain-containing protein 3
PAD4: Peptidyl arginine deiminase 4
PLA2R: Phospholipase A2 receptor
PR3: Proteinase 3
PRR: Pattern recognition receptor
SASP: Senescence-associated secretory phenotype
SLE: Systemic lupus erythematosus
STING: Stimulator of interferon genes
TCR: T cell receptor
TEC: Tubular epithelial cell
TGF-β: Transforming growth factor-beta
TLR: Toll-like receptor
TLS: Tertiary lymphoid structure
aHUS: Atypical hemolytic uremic syndrome
